# Prevalence and patterns of mutations in *RAS/RAF/MEK/ERK/MAPK* signaling pathway in colorectal cancer in North Africa

**DOI:** 10.1186/s12885-022-10235-w

**Published:** 2022-11-07

**Authors:** Meryem Jafari, Abdelilah Laraqui, Walid Baba, Soukaina Benmokhtar, Sara El Zaitouni, Abdelmounaim Ait Ali, Ahmed Bounaim, Mountassir Moujahid, Rachid Tanz, Tarik Mahfoud, Yassir Sbitti, Hicham El Annaz, Rachid Abi, Mohamed Rida Tagajdid, Safae El Kochri, Idriss Amine Lahlou, Houda El Hsaini, Lamiae Belayachi, Abdelaziz Benjouad, Mohammed Ichou, Amina En-Nya, Khalid Ennibi

**Affiliations:** 1 Sequencing Unit, Laboratory of Virology, Center of Virology, Infectious and Tropical Diseases, Faculty of Medicine and Pharmacy, Mohammed V Military Teaching Hospital, Mohammed V University in Rabat, Rabat, Morocco; 2 Laboratory of Biology of Human Pathologies, Department of Biology, Faculty of Sciences, Genomic Center of Human Pathologies, Mohammed V University in Rabat, Rabat, Morocco; 3 Department of Digestive Surgery, Faculty of Medicine and Pharmacy, Mohammed V Military Hospital, Mohammed V University in Rabat, Rabat, Morocco; 4 Department of Medical Oncology, Faculty of Medicine and Pharmacy, Mohammed V Military Teaching Hospital, Mohammed V University in Rabat, Rabat, Morocco; 5 International Faculty of Dental Medicine, College of Health Sciences, International University in Rabat, Rabat, Morocco; 6 Center of Virology, Infectious and Tropical Diseases, Faculty of Medicine and Pharmacy, Mohammed V Military Teaching Hospital, Mohammed V University in Rabat, Rabat, Morocco

**Keywords:** *KRAS*, *NRAS*, And *BRAF* mutations, Colorectal cancer, Lifestyle factors, North Africa

## Abstract

**Background:**

Our review discuss (i) the findings from analyzed data that have examined *KRAS*, *NRAS* and *BRAF* mutations in patients with colorectal cancer (CRC) in North Africa and to compare its prevalence with that shown in other populations and (ii) the possible role of dietary and lifestyle factors with CRC risk.

**Methods:**

Using electronic databases, a systematic literature search was performed for the *KRAS*, *NRAS*, and *BRAF* mutations in CRC patients from Morocco, Tunisia, Algeria and Lybia.

**Results:**

Seventeen studies were identified through electronic searches with six studies conducted in Morocco, eight in Tunisia, two in Algeria, and one in Libya. A total of 1843 CRC patients were included 576 (31.3%) in Morocco, 641 (34.8%) in Tunisia, 592 (32.1%) in Algeria, and 34 (1.8%) in Libya. Overall, the average age of patients was 52.7 years old. Patients were predominantly male (56.6%). The mutation rates of *KRAS*, *NRAS* and *BRAF* were 46.4%, 3.2% and 3.5% of all patients, respectively. A broad range of reported *KRAS* mutation frequencies have been reported in North Africa countries. The *KRAS* mutation frequency was 23.9% to 51% in Morocco, 23.1% to 68.2% in Tunisia, 31.4% to 50% in Algeria, and 38.2% in Libya. The G12D was the most frequently identified *KRAS* exon 2 mutations (31.6%), followed by G12V (25.4%), G13D (15.5%), G12C (10.2%), G12A (6.9%), and G12S (6.4%). G12R, G13V, G13C and G13R are less than 5%. There are important differences among North Africa countries. In Morocco and Tunisia, there is a higher prevalence of G12D mutation in *KRAS* exon 2 (≈50%). The most frequently mutation type in *KRAS* exon 3 was Q61L (40%). A59T and Q61E mutations were also found. In *KRAS* exon 4, the most common mutation was A146T (50%), followed by K117N (33.3%), A146P (8.3%) and A146V (8.3%).

**Conclusion:**

*KRAS* mutated CRC patients in North Africa have been identified with incidence closer to the European figures. Beside established anti-CRC treatment, better understanding of the causality of CRC can be established by combining epidemiology and genetic/epigenetic on CRC etiology. This approach may be able to significantly reduce the burden of CRC in North Africa.

## Introduction

Colorectal cancer (CRC) is the third most commonly occurring cancer in men and the second most commonly occurring cancer in women. According to GLOBOCAN 2020 data, there are 1.15 million new cases of colon cancer, 0.7 million new cases of rectal cancers, and 50,000 new cases of anal cancer in 2020 globally [1]. With continuous progress, these numbers are predicted to increase to 1.92 million, 1.16 million, and 78,000 in 2040, respectively [2]. Globally it is one of the cancers whose incidence is increasing comprising 11% of all cancer diagnoses [3]. Over the world, the risk of CRC is highest and 3–4 times more common in countries with a very high human development index (HDI) than in countries with a low HDI [4]. CRC is considered one of the clearest markers of epidemiological and nutritional transition in societies undergoing socioeconomic development and transition to a lifestyle more typical of industrialized countries. Moreover, the data suggest the existence of a threshold at which CRC incidence stabilizes or declines according to the HDI [5].

Studies of CRC in the North of Africa (including Morocco, Algeria, Tunisia, and Libya) have shown rising trend in incidence rate. It was 7.1/100,000 [6]. When compared with sub-Saharan Africa (SSA), North Africa had the highest age-standardized incidence rates (ASR) of 8.66, while SSA had an ASR of 5.91 [7]. Libya reports the highest ASR for CRC with an incidence closer to the European figures. The ASR per 100,000 for CRC was 17.5 and 17.2 for males and females, respectively [8]. CRC ASR in Northern Tunisia was of 12.4 in 2009 (13.6 against 11.1, respectively, in men and women). This incidence increased during the period 1994–2009, from 6.4/100,000 in 1994 to 12.4/100,000 in 2009 [9]. CRC in young patients is more common in North Africa countries with a proportion of 11% in Morocco and much higher in the Middle East with 46% in UAE [10] and 25% in Egypt [11].

CRC is a complex and genetically heterogeneous disease with involving in oncogene and tumor suppressor genes, as well as genes involved in DNA damage recognition and repair. CRC includes also different categories of tumors based on their specific spectrum of mutations and molecular phenotype, driving various oncogenic signaling pathways [12]. The *RAS/RAF/MEK/ERK/MAPK* is the most well-known pathway in the pathogenesis of CRC. Mutations in genes of this pathway have been reported in about 50% of patients with CRC [13]. *RAS* and *BRAF* are members of the *RAS/RAF/MEK/ERK/MAPK* pathway which mediates cellular response to growth signals. Both *RAS* and *RAF* are members of multi-gene families and there are three *RAS* members (*KRAS*, *NRAS* and *HRAS*) and three *RAF* members (*BRAF*, *RAF1* (a.k.a c-Raf) and *ARAF*). The *KRAS* proto-oncogene (locus 1p13.2) is the most commonly mutated RAS family member (75% of *RAS* mutations). The *KRAS* proto-oncogene encodes a protein (p21-ras) belonging to the family of GTP/GDP-binding proteins with GTPase activity and is involved in the transduction of mutagenic signals. *KRAS* gene mutations occur commonly in colon [14, 15]. The *NRAS* proto-oncogene (locus 1p13.2) is also a member of the *RAS* gene family which encodes proteins involved in signal transmission in cells and participates in the regulation of cell growth. *NRAS* gene mutations is associated with of CRC tumors [14, 15]. The *BRAF* gene on chromosome 7 (7q34) encodes for a serine/threonine protein kinase) that sends signals from outside of the cell to the nucleus that in turn drives the growth of a cell. *BRAF* is a downstream target of *RAS*, playing a pivotal role in the MAPK/ERK signaling pathway. Mutations of *BRAF* are found in CRCs [16] and are associated with a poor prognosis in stage II, III, and IV [17].

This systematic review aims to discuss (i) the findings from analyzed data that have examined *KRAS*, *NRAS* and *BRAF* mutations in patients with CRC in North Africa and to compare its prevalence with that shown in other populations and (ii) the possible role of dietary and lifestyle factors with CRC risk.

## Materials and methods

We included articles that described the prevalence of *KRAS*, *NRAS*, or *BRAF* gene mutations in CRC patients in North Africa. Pub Med, Science Direct and Google Scholar were searched up to December 2021 for eligible studies using the following keywords: “colorectal cancer”, or “colon cancer”, or “rectum cancer”, or “*RAS*”, or “*KRAS*”, or “*NRAS*”, or “*BRAF*”, or “mutation”, or “oncogenic mutation”, or “oncogenic driver mutation”, or “activating mutation”, or “prevalence”, or “rate”, or “incidence”, or “frequency”.

An additional literature search was also conducted using North Africa and specific country names belonging to the considered region and any other variant names for any of North Africa countries (ex: Mediterranean countries, Maghreb, Arab population). We manually checked reference lists of the included studies and relevant reviews to identify additional studies. We also searched relevant abstracts reported in the most important multi-disciplinary societies of medical oncology such as the American Society of Clinical Oncology (ASCO) meetings to identify unpublished studies.

Eligible analytical study designs were prospective or retrospective cohort studies, cross-sectional, and case–control studies and were conducted in CRC patients. The study must give information on the prevalence of *KRAS*, *NRAS*, and *BRAF* mutations in a specific or selected coding region of *KRAS*, *NRAS* genes (exon 2: codon 12 and 13; exon 3: codon 59 and 61; exon 4: codon 117 and 146) and *BRAF* (exon 15). Studies published as original articles on human subjects, evaluating *KRAS, NRAS,* and *BRAF* mutation status and recording clinico-pathological features in CRC patients, and written in English, were included for analysis.

The following information data were abstracted from each of the eligible studies: 1) characteristics of the study (including publication year, country where the study was conducted, study type, sample size, number of subjects with mutation results); 2) characteristics of study participants (including gender, age); 3) characteristics of study outcomes (including mutation detection assay, type of mutation, mutation exon/codon numbers, mutation prevalence, whether mutation data were from primary or metastatic tumor, and whether mutation data were obtained from tissue or liquid biopsy using plasma); 4) clinicopathological characteristics (including disease stage, T stage, metastasis status, tumor site, tumor differentiation and tumor histology).

Letters, comments, and review articles or meta-analysis without original data, were excluded from analysis.

## Results

The search of the databases yielded 17 relevant references which are closely related to defining the inclusion criteria and was included in this review. The retrieved articles describe studies conducted in Morocco (*n* = 6) [18–23], Tunisia (*n* = 8) [24–31], Algeria (*n* = 2) [32, 33], and Libya (*n* = 1) [34]. A total of 1843 patients with CRC were included for our analysis, including 576 (31.3%) in Morocco [18–23], 641 (34.8%) in Tunisia [24–31], 592 (32.1%) in Algeria [32, 33], and 34 (1.8%) in Libya [34]. The average age of patients was 52.7 years old [18–20, 22–28, 31–33], with 45.1% (260/577) [18, 20, 26, 29, 34] of patients diagnosed above the age of 50 years. Male patients were predominant in all of the considered studies, accounting for 56.6% (960/1697) [18–20, 22–26, 28, 29, 31–34].

From the specimens where tumor site information was available, 31.4% (344/1095) of them were in the rectum [22, 23, 26, 28, 29, 31–34], 30.1% (188/625) were in the right colon [18, 22, 24, 25, 28, 31], and 56.5% (353/625) in the left colon [18, 22, 24, 25, 28, 31]. The predominant histopathological type of CRC is adenocarcinoma (82.2%, 374/455) [18, 20, 25]. Almost half (58%, 302/521) of the tumors were reported to be in stage III and IV [18, 23, 24, 27, 33]. Baseline characteristics and clinico-pathologic of enrolled studies are summarized in Table 1.

**Table 1:** Baseline characteristics and clinico-pathologic of enrolled studies from North Africa countries

Country Author [reference]	Sample size	Mean age Years (± SD)	Women/Men N (%)	Tumor site N (%)			Disease stages Stage: N (%)
				**Colon**		**Rectum**	
				**Right**	**Left**		
**Morocco**							
El Agy et al. [18]	210	55.6 (± 14.3)	97 (46.2%)/113 (53.8%)	67 (31.9%)	143 (68.1%)	na	I-II: 126 (60%), III-IV: 84 (40%)
Houssaini et al. [19]	51	59,8	24 (48.1%)/27 (52.9%)	na	na	na	na
Dehbi et al. [20]	114	55.4	50 (43.9%)/64 (56.1%)	na	na	na	na
Jadda et al. [21]	47	na	na	na	na	na	na
Marchoudi et al. [22]	92	62.3	38 (41.3%)/54 (58.7%)	15 (16.3%)	34 (37%)	12 (13.0%)	na
Bennani et al. [23]	62	53	31 (50%)/31 (50%)	na	na	28 (44.4%)	II: 22 (35.5%), III: 26 (41.9%), IV: 14 (22.6%)
**Tunisia**							
Ounissi et al. [24]	96	62.4	36 (37.5%)/60 (62.5%)	25 (26.1%)	71 (73.9%)	na	I-II: 32 (33.3%), III-IV: 64 (66.6%)
Jouini et al. [25]	131	56.1 (± 12.6)	56 (42.7%)/75 (57.3%)	37 (78.7%)	75 (92.6%)		na
Chaar et al. [26]	167	57	83 (49.7%)/84(50.3%)	93 (55.7%)		74 (44.3%)	I-II: 30 (17.9%), III-IV: 137 (82%)
Aissi et al. [27]	51	48.5	na	na	na	na	II: 13 (48.1%), III: 12 (44.4%), IV: 2 (7.4%)
Ouerhani et al. [28]	48	61 (± 13)	25 (52%)/23 (47.1%)	21 (43.7%)	18 (37.5%)	9 (18.8%)	na
Sammoud et al. [29]	52	na	23 (44.2%)/29 (55.8%)	25 (48.1%)		27 (51.9%)	na
Bougatef et al. [30]	48	na	na	na	na	na	na
Bougatef et al. [31]	48	61 (± 13)	25 (52%)/23 (47.1%)	22 (45.8%)	15 (31.3%)	9 (18.8%)	na
**Algeria**							
Mazouzi et al. [32]	490	52.6	190 (38.8%)/300 (61.2%)	331 (67.6%)		141 (28.8%)	na
Boudida-Berkane K et al. [33]	102	48(47.1%) < 50 54(52.9%) > 50	44 (43.2%)/58 (56.8%)	66 (64.7%)		36 (35.3%)	III: 46 (45%), IV: 56 (55%)
**Lybia**							
Abudabous et al. [34]	34	na	15 (44.1%)/19 (55.9%)	11 (32.35%)	11 (32.35%)	8 (23.5%)	na

*na* not available

Among the 17 studies, 13 (76.5%) [18–25, 27, 30–33] used FFPE tumor samples for *KRAS*, *NRAS* and *BRAF* mutation analysis. Both exon 2, 3, and 4 mutations of *KRAS* and *NRAS* genes were analyzed in 23.5% (4/17) [18, 19, 25, 32] of North Africa series, exon 2, 3 and 4 mutations of *KRAS* gene were evaluated in one study (5.9%, 1/17) [33], specific *KRAS* exons (exon 2 and/or exon3) were assessed in 58.8% (10/17) [20–24, 26–30] and *NRAS* exons (exon 2 and 3) in 11.8% (2/17) investigations [20, 24]. *KRAS* mutations in exon 2 codon 12 and 13 have been assessed in the most of the studies (94.1%, 16/17) [18–30, 32–34]. *BRAF* gene mutation was analyzed in 41.2% (7/17) studies [18, 19, 21–23, 31, 33].

Different molecular methods were used for *KRAS*, *NRAS* and *BRAF* mutations screening. Sequencing assay was broadly used, as it was used in 64.70% (11/17) studies [21, 23, 26–34]. Other methodologies described in the considered studies include Pyrosequencing [18, 20, 25], array based techniques Mass ARRAY [19, 22], amplification-refractory mutation system-PCR (ARMS-PCR) [24], Allele-specific PCR [32], End-point genotyping [22], High Resolution Melting [30] and Denaturating High Performance Liquid Chromatography [30]. The details of studies examining *KRAS*, *NRAS* and *BRAF* genes in North Africa are presented in Table 2.

**Table 2:** Details of studies examining *KRAS*, *NRAS* and *BRAF* genes in North Africa

Country Author [reference]	Materials	KRAS			NRAS			BRAF	Molecular analysis
		**Exon 2**	**Exon 3**	**Exon 4**	**Exon 2**	**Exon 3**	**Exon 4**	**Exon 15**	
**Morocco**									
El Agy et al. [18]	FFPE	+	+	+	+	+	+	-	Pyrosequencing
Houssaini et al. [19]	FFPE	+	+	+	+	+	+	-	MassARRAY
Dehbi et al. [20]	FFPE	+	+	-	+	+	-	-	Pyrosequencing
Jadda et al. [21]	FFPE	+	-	-	-	-	-	+	Sequencing
Marchoudi et al. [22]	FFPE	+	-	-	-	-	-	+	KRAS array, End-point genotyping
Bennani et al. [23]	FFPE	+	-	-	-	-	-	+	HRM and sequencing
**Tunisia**									
Ounissi et al. [24]	FFPE	+	-	-	+	+	-	-	PCR
Jouini et al. [25]	FFPE	+	+	+	+	+	+	-	Pyrosequencing
Chaar et al. [26]	Tumoral mucosa	+	+	-	-	-	-	-	PCR- SSCP, Sequencing
Aissi et al. [27]	FFPE	+	-	-	-	-	-	-	Sequencing
Ouerhani et al. [28]	Fresh tumour or FFPE	+	-	-	-	-	-	-	Sequencing
Sammoud et al. [29]	Normal mucosa and tumoral mucosa of epithelial tissue	+	-	-	-	-	-	-	Sequencing
Bougatef et al. [30]	FFPE	+	-	-	-	-	-	-	Sequencing, HRM, DHPLC
Bougatef et al. [31]	FFPE	-	-	-	-	-	-	+	Sequencing
**Algeria**									
Mazouzi et al. [32]	FFPE	+	+	+	+	+	+	-	ASPCR-Sequencing
Boudida-Berkane K et al. [33]	FFPE	+	+	+	-	-	-	+	Sequencing
**Lybia**									
Abudabous et al. [34]	Frozen tissue	+	-	-	-	-	-	-	Sequencing

+ genetic analysis realized, - genetic analysis not realized, *HRM* High Resolution Melting, *DHPLC* Denaturating High Performance Liquid Chromatography, *ASPCR* Allele-specific PCR

The prevalence of *KRAS*, *NRAS* and *BRAF* mutations has been reported in 16 [18–30, 32–34], 6 [18–20, 24, 25, 32], and 7 [18, 19, 21–23, 31, 33] of the 17 included studies, respectively. In total, *KRAS* mutations were most frequently detected among North Africa patients with CRC, accounting for 41.7% (749/1795) [18–30, 32–34]. Tunisia highlights a wide range of *KRAS* mutations rates, ranging from 23.1% (12/52) [29] to 68.2% (88/129) [25], as compared with other countries from the region. Overall, *KRAS* Mutations were distributed among the different exons as follows: 95.9% (718/749) exon 2 [18–27, 29, 30, 32–34], 2.7% (13/481) exon 3 [18, 20, 25, 32], and 2.9% (14/481) exon 4 [18–20, 25] (Table3). Among mutations in exon 2, 79.7% (94/118) had single mutations in codon 12 [18, 22, 27, 34] and 20.3% (24/118) had point mutations in codon 13 [18, 22, 27, 34] (Table 4).

**Table 3:** Prevalence of *KRAS, NRAS* and *BRAF* mutations in North Africa

Country Author [reference]	Gene frequency n/N (%)			Exon frequency n/N (%)						
	**KRAS**	**NRAS**	**BRAF**	**KRAS Exon2**	**KRAS Exon3**	**KRAS Exon4**	**NRAS Exon2**	**NRAS Exon3**	**NRAS Exon4**	**BRAF Exon15**
**Morocco**										
El Agy et al. [18]	77/210 (36.7%)	6/210 (2.9%)	0/210 (0%)	68 (83.3%)	1 (1.3%)	8 (10.4%)	2 (33.3%)	4 (66.7%)	na	na
Houssaini et al. [19]	22/51 (43.1%)	1/51 (2%)	2/51 (3.9%)	26^a^ (92.8%)	na	2^a^ (7.1%)	na	na	na	2 (3.9%)
Dehbi et al. [20]	49/114 (43%)	6/114 (5.3%)	na	45 (91.8%)	3 (6.1%)	1 (2.04%)	na	6 (5.3%)	na	na
Jadda et al. [21]	24/47 (51%)	na	0/47 (0%)	24 (51%)	na	na	na	na	na	0 (0%)
Marchoudi et al. [22]	22/92 (23.9%)	na	5/92 (5.4%)	22/92 (23.9%)	na	na	na	na	na	5 (5.4%)
Bennani et al. [23]	18/62 (29%)	na	1/62 (1.6%)	18 (38.7%)	na	na	na	na	na	1 (1.6%)
**Tunisia**										
Ounissi et al. [24]	40/96 (41.7%)	7/96 (7.3%)	na	47^b^ (100%)	na	na	5^c^ (62.5%)	3^c^ (37.5%)	0^c^ (0%)	na
Jouini et al. [25]	88/129 (68.2%)	9/129 (6.9%)	na	84 (95.4%)	1 (1.1%)	3 (3.4%)	3 (33.3%)	4 (44.4%)	2 (22.2%)	na
Chaar et al. [26]	52/167 (31.1%)	na	na	52 (31.1%)	na	na	na	na	na	na
Aissi et al. [27]	16/51 (31.5%)	na	na	16 (31.5%)	na	na	na	na	na	na
Ouerhani et al. [28]	17/48 (35.41%)	na	na	ni	na	na	na	na	na	na
Sammoud et al. [29]	12/52 (23.1%)	na	na	12 (23.1%)	na	na	na	na	na	na
Bougatef et al. [30]	22/48 (45.83%)	na	na	22 (45.83%)	na	na	na	na	na	na
Bougatef et al. [31]	na	na	4/48 (8.3%)	Na	na	na	na	na	na	4 (8.3%)
Algeria										
Mazouzi et al. [32]	245/490 (50%)	6/490^d^ (1.2%)	na	237 (96.7%)	8 (3.3%)	na	na	na	na	Na
Boudida-Berkane K et al. [33]	32/102 (31.4%)	na	5/102 (4.9%)	32 (31.4%)	na	na	na	na	na	5 (4.9%)
**Lybia**										
Abudabous et al. [34]	13/34 (38.2%)	na	na	13 (38.2%)	na	na	na	na	na	na

*na* not available

^a^In about 22 Patients with a KRAS mutations, 28 mutations were detected in exon 2 and exon 4 (Some patients have concomitant mutations)

^b^In a total of 40 cases, we had 47 mutations in KRAS exon 2 (7/40 patients had 2 concomitant mutations in exon 2)

^c^One of the 7 patients with NRAS mutation showed a double mutation in NRAS

^d^Rare mutations in the NRAS gene

**Table 4:** *KRAS* and *NRAS* exons mutations subtypes detected in North Africa

Country Author [reference]	KRAS Exon2		KRAS Exon3		KRAS Exon4		NRAS Exon2		NRAS Exon3		NRAS Exon4	
	**Codon 12**	**Codon 13**	**Codon 59**	**Codon 61**	**Codon 117**	**Codon 146**	**Codon 12**	**Codon 13**	**Codon 59**	**Codon 61**	**Codon 117**	**Codon 146**
**Morocco**												
El Agy et al. [18]	52/68 (76.5%)	16/68 (23.5%)	na	1/77 (1.3%)	3/8 (37.5%)	5/8 (62.5%)	2/6 (33.3%)	na	na	4/6 (66.7%)	na	na
Houssaini et al. [19]	na	na	na	Na	na	Na	na	na	na	na	na	na
Dehbi et al. [20]	37/45 (82.2%)	8/45 (17.8%)	1/3 (33.3%)	2/3 (66.7%)	na	1/49 (2.04%)	na	na	na	6/114 (5.3%)	na	na
Jadda et al. [21]	21/24 (87.5%)	3/24 (12.5%)	na	na	na	na	na	na	na	na	na	na
Marchoudi et al. [22]	18/22 (81.8%)	4/22 (18.2%)	na	na	na	na	na	na	na	na	na	na
Bennani et al. [23]	15/18 (83.3%)	3/18 (16.7%)	na	na	na	na	na	na	na	na	na	na
**Tunisia**												
Ounissi et al. [24]	40/47 (85%)	7/47 (15%)	na	na	na	na	na	na	na	3/8 (37.5%)	na	0/8 (0%)
Jouini et al. [25]	72/84 (85.7%)	12/84 (14.3%)	na	1/88 (1.1%)	1/3 (33.3%)	2/3 (66.7%)	3/9 (33.3%)	na	na	4/9 (44.4%)	1/2 (50%)	1/2 (50%)
Chaar et al. [26]	43/52 (82.7%)	9/52 (17.3%)	na	na	na	na	na	na	na	na	na	Na
Aissi et al. [27]	13/16 (81.2%)	3/16 (18.7%)	na	na	na	na	na	na	na	na	na	na
Ouerhani et al. [28]	na	na	na	na	na	na	na	na	na	na	na	na
Sammoud et al. [29]	10/12 (83.3%)	2/12 (16.7%)	na	na	na	na	na	na	na	na	na	na
Bougatef et al. [30]	na	na	na	na	na	na	na	na	na	na	na	na
Bougatef et al. [31]	na	na	na	na	na	na	na	na	na	na	na	na
**Algeria**												
Mazouzi et al. [32]	182/237 (76.8%)	55/237 (23.2%)	na	na	na	na	na	na	na	na	na	na
Boudida-Berkane K et al. [33]	29/32 (90.6%)	3/32 (9.4%)	na	na	na	na	na	na	na	na	na	na
**Lybia**												
Abudabous et al. [34]	12/13 (92.3%)	1/13 (7.7%)	na	na	na	na	na	na	na	na	na	na

*na* not available

As seen in Table 5, the G12D was the most frequently identified exon 2 mutations (31.6%, 137/433) [18, 20–27, 29, 33, 34], followed by G12V (25.4%, 110/433) [18, 20–26, 29, 33, 34], G13D (15.5%, 67/433) [18, 20–27, 29, 33, 34], G12C (10.2%, 44/433) [18, 20–27, 29, 33, 34], G12A (6.9%, 30/433) [18, 20–22, 24, 27, 29, 33], G12S (6.4%, 28/433) [20, 23–25, 27, 33]. G12R, G13V, G13C and G13R are less than 5% [18, 20, 21, 24–26]. However, there are important differences among North Africa countries. In Morocco and Tunisia there is a higher prevalence of G12D mutation (50%) [22, 27]. The most frequently mutation type in exon 3 was Q61L (40%, 2/5) [18, 20]. A59T [20] and Q61E [25] mutations were also found in our review. In exon 4, the most common mutation was A146T (50%, 6/12) [18, 20, 25], followed by K117N (33.3%, 4/12) [18, 25], A146P (8.3%, 1/12) [18] and A146V (8.3%, 1/12) [18].

**Table 5:** Frequency and distribution of *KRAS, NRAS* and *BRAF* mutations in different North of Africa countries

Gene	Exon	Codon	Amino acid	Nucleotide	Protein	Morocco	Tunisia	Algeria	Lybia
KRAS	2	12	G12D	c.35G > A	p.Gly12Asp	19 (24.7%) [18] 7 (14.3%) [20] 9 (50%) [23] 7 (29.2%) [21] 11(50%) [22]	12(25.5%) [24] 17(19.3%) [25] 22(42.3%) [26] 8(50%) [27] 5(41.7%) [29]	14(43.7%) [33]	6(46.1%) [34]
KRAS	2	12	G12V	c.35G > T	p.Gly12Val	17 (22.1%) [18] 18 (36.7%) [20] 3(16.7%) [23] 10(41.7%) [21] 2(9.1%) [22]	12(25.5%) [24] 25(28.4%) [25] 13(25%) [26] 3(25%) [29]	3(9.4%) [33]	4(30.8%) [34]
KRAS	2	12	G12C	c.34G > T	p.Gly12Cys	9 (11.7%) [18] 4 (8.2%) [20] 1 (4.2%) [21] 2(11.1%) [23] 3(13.6%) [22]	3(6.4%) [24] 12(13.6%) [25] 4(7.7%) [26] 1(6.2%) [27] 1(8.3%) [29]	2(6.2%) [33]	2(15.4%) [34]
KRAS	2	12	G12S	c.34G > A	p.Gly12Ser	4 (8.2%) [20] 1(5.6%) [23]	2(4.3%) [24] 17(19.3%) [25] 2(12.5%) [27]	2(6.2%) [33]	na
KRAS	2	12	G12A	c.35G > C	p.Gly12Ala	5 (6.5%) [18] 3(6.1%) [20] 1 (4.2%) [21] 2(9.1%) [22]	8(17%) [24] 2(12.5%) [27] 1(8.3%) [29]	8(25%) [33]	na
KRAS	2	12	G12R	c.34G > C	p.Gly12Arg	2 (2.6%) [18] 1(2.0%) [20] 2 (8.33%) [21]	3(6.4%) [24] 1(1.1%) [25] 4(7.7%) [26]	na	na
KRAS	2	13	G13D	c.38G > A	p.Gly13Asp	13(16.9%) [18] 7 (14.3%) [20] 3 (12.5%) [21] 3(16.7%) [23] 4(18.2%) [22]	7(14.9%) [24] 12(13.6%) [25] 9(17.3%) [26] 3(18.7%) [27] 2(16.7%) [29]	3(9.4%) [33]	1(7.7%) [34]
KRAS	2	13	G13V	c.38G > T	p.Gly13Val	2(2.6%) [18]	na	na	na
KRAS	2	13	G13R	c.37G > C	p.Gly13Arg	1(1.3%) [18]	na	na	na
KRAS	2	13	G13C	c.37G > T	p.Gly13Cys	1(2.0%) [20]	na	na	na
KRAS	3	59	A59T	c.175G > A	p.Ala59Thr	1(2.0%) [20]	na	na	na
KRAS	3	59	A59G	c.176C > G	p.Ala59Gly	na	na	na	na
KRAS	3	61	Q61H	c.183A > C	p.Gln61His	na	na	na	na
KRAS	3	61		c.183A > G		1(2.0%) [20]	na	na	na
KRAS	3	61	Q61L	c.182A > T	p.Gln61Leu	1(1.3%) [18] 1(2.0%) [20]	na	na	na
KRAS	3	61	Q61R	c.182A > G	p.Gln61Arg	na	na	na	na
KRAS	3	61	Q61E	c.181 C > G	p.Gln61Glu	na	1(1.1%) [25]	na	na
KRAS	4	117	K117N	c.351A > C	p.Lys117Asn	3(3.9%) [18]	1(1.1%) [25]	na	na
KRAS	4	146	A146T	c.436G > A	p.Ala146Thr	3(3.9%) [18] 1(2.0%) [20]	21(2.3%) [25]	na	na
KRAS	4	146	A146P	c.436G > C	p.Ala146Pro	1(1.3%) [18]	na	na	na
KRAS	4	146	A146V	c.437C > T	p.Ala146Val	1(1.3%) [18]	na	na	na
NRAS	2	12	G12S	c.34G > A	p.Gly12Ser	na	1(11.1%) [25]	na	na
NRAS	2	12	G12C	c.34G > T	p.Gly12Cys	na	na	na	na
NRAS	2	12	G12R	c.34G > C	p.Gly12Arg	na	na	na	na
NRAS	2	12	G12D	c.35G > A	p.Gly12ASP	2(33.3%) [18]	2(22.2%) [25]	na	na
NRAS	2	12	G12V	c.35G > T	p.Gly12Val	na	na	na	na
NRAS	2	12	G12A	c.35G > C	p.Gly12Ala	na	na	na	na
NRAS	2	13	G13S	C.37G > A	p.Gly13Ser	na	na	na	na
NRAS	2	13	G13C	c.37G > T	p.Gly13Cys	na	na	na	na
NRAS	2	13	G13R	c.37G > C	p.Gly12Arg	na	na	na	na
NRAS	2	13	G13D	c.38G > A	p.Gly13Asp	na	na	na	na
NRAS	2	13	G13V	c.38G > T	p.Gly13Val	na	na	na	na
NRAS	2	13	G13A	c.38G > C	p.Gly13Ala	na	na	na	na
NRAS	3	59	A59T	c.175G > A	p.Ala59Thr	na	na	na	na
NRAS	3	59	A59G	c.176 C > G	p.Ala59Gly	na	na	na	na
NRAS	3	61	Q61K	c.181C > A	p.Gln61Lys	2(33.3%) [18] 3(50%) [20]	1(11.1%) [25]	na	na
NRAS	3	61		c.181A > G		1(16.7%) [20]	na	na	na
NRAS	3	61	Q61R	c.182A > G	p.Gln61Arg	2(33.3%) [20]	na	na	na
NRAS	3	61	Q61L	c.182A > T	p.Gln61Leu	2(33.3%) [18]	na	na	na
NRAS	3	61	Q61H	c.183A > T	p.Gln61His	na	3(33.3%) [25]	na	na
NRAS	3	61	Q61E	c.181C > G	p.Gln61Glu	na	na	na	na
NRAS	4	117	K117N	c.351G > C	p.Lys117Asn	na	1(11.1%) [25]	na	na
NRAS	4	117	K117N	c.351G > T	p.Lys117Asn	na	na	na	na
NRAS	4	146	A146T	c.426G > A	p.Ala146Thr	na	1(11.1%) [25]	na	na
NRAS	4	146	A146V	c.437C > T	p.Ala146Val	na	na	na	na
BRAF	15	600	V600E	1799 T > A	p.Val600Glu	2(3.9%) [19] 5(5.4%) [22] 1(1.6%) [23]	4(8.3%) [31]	5(4.9%) [33]	na

*na* not available

*NRAS* total mutations were identified in 3.2% (35/1090) [18–20, 24, 25, 32] tumor samples. Morocco highlights a wide range of *NRAS* mutations rates, ranging from 2% (1/51) to 5.3% (6/114) [19, 20]. A higher prevalence of *NRAS* mutations has been reported in Tunisia (7.3%, 7/96) [24]. Overall, the common mutation site of *NRAS* gene was located in exons 2 and 3, with 28.6% (10/35) [18, 24, 25] and 48.6% (17/35) [18, 20, 24, 25] of CRC patient (Table 3). *NRAS* mutations were more common in codon 61, accounting for 48.6% (17/35)[18, 20, 24, 25] (Table 4). The most common mutations were G12D in exons 2 and Q61K in exon 3 and accounted for 40% (4/10) [18, 25] and 35.3% (6/17) [18, 20, 25] of patients with CRC, respectively (Table 5).

The total mutation frequency in the *BRAF* gene was 2.8% (17/602) [18, 19, 21–23, 31, 33]. The highest frequency of *BRAF* mutations was found in Tunisia and reported in 8.3% (4/48) of patients with CRC [31]. The overall frequency of the *BRAF* V600E mutation was 2.8% (17/602) [18, 19, 21–23, 31, 33] (Tables 3 and 5).

## Discussion

CRC is a pathologically and clinically heterogeneous malignancy. Phenotypic and molecular characteristics of CRC represents a significant key step in defining diagnosis, prognosis and treatment predictive value both in localized and in the setting of CRC [35]. Mutation detection in any of the genes involved in the *RAS*/*RAF*/*MEK*/*ERK/MAPK* pathway has been used to predict the outcomes of *EGFR*-targeted therapy for CRC. *KRAS* mutations were found in ≈33% of in the COSMIC dataset comprising 75,000 tested specimens samples. In other datasets based on small sample sizes (< 500), *KRAS* was found mutated in ≈40%-45% CRCs. Notably however, the private Foundation Medicine dataset comprising 13,336 colorectal samples reports a *KRAS* mutation frequency of ≈50% [36]. According to the COSMIC database (Catalogue of Somatic Mutations in Cancer, http://cancer.sanger.ac.uk/cosmic), mutations at codon 12 (G12A, G12V, G12S, G12R, G12C, G12D) are the most prominent (> 90%), followed by those affecting codon 13 (G13D, G13C), codon 61 (Q61L, Q61R, Q61H), codon 146 (A146T, A146V, A146P), and codon 117 (K117N). *NRAS* mutations are found in 5%-10% of CRC. *NRAS* is mutated in the same codon of *KRAS* particularly in exon 2 (3%-5%) and exon 3 (2%-6%). *BRAF* mutations are identified in about 8%-12% of CRC patients and 90% of all identified mutations are a T1799A transversion in exon 15, which results in a valine amino acid substitution (V600E) [37].

In our review, *KRAS* mutation was detected in the CRC tumor of 41.7% of the patients among whom 95.9% had a single mutation at exon 2, 2.7% at exon 3, and 2.9% at exon 4. Among mutations in *KRAS* exon 2, 79.7% of cases had single mutations in codon 12 and 20.3% of cases in codon 13. The G12D was the most frequently identified exon 2 mutations (31.6%), followed by G12V, G13D, G12C, G12A, and G12S. The most frequently mutation type in exon 3 was Q61L (40%). In exon 4, the most common mutation was A146T, K117N, A146P and A146V. *NRAS* total mutations were identified in 3.2% tumor samples. The common mutation site of *NRAS* gene was located in exons 2 and 3, with 28.6% and 48.6% of CRC patient. *NRAS* mutations were more common in codon 61, accounting for 48.6%. The most common mutations were G12D in exon 2 and Q61K in exon 3 and accounted for 40% and 35.3% of CRC patients, respectively. In *BRAF* gene, the overall frequency of the *BRAF* V600E mutation was 2.8%.

Our review of mutation prevalence among CRC patients in North Africa identified a significant difference in prevalence of both *RAS* (*KRAS* and *NRAS*) and *BRAF* mutations. Interestingly, our finding showed that the *KRAS* mutation rate was different with more investigation that therefore the geographic location and ethnicity/race can impact on the prevalence of *KRAS* mutation in CRC patients. The *KRAS* mutation frequency was 23.9% to 51% in a Moroccan population [18–23], 23.1% to 68.2% in a Tunisian population [24–30], 31.4% to 50% in an Algerian population [32, 33], and 38.2% in a Libyan population [34]. This broad range of reported *KRAS* mutation frequencies may be related with diverse ethnic background. Arabs and Berbers make up the overwhelming majority of the population throughout the North Africa today. However, throughout its history, North Africa has been the site of invasions and migratory waves of ethnic groups such as Phoenicians, Romans, Vandals, Arabs, Ottomans and Europeans.

Worldwide, a persistent finding in the literature is the substantial variation in *KRAS* mutation frequency between race/ethnic groups. In Nigerian, the frequency of CRC with *KRAS* mutations is 21% [38]. In Asian populations, *KRAS* mutations vary from 37.9% (Japan) to 52.7% (China) [39, 40], while Middle Eastern population reported frequencies of 32% (Saudi Arabia) and 48% (Iraq) [41, 42]. Data from USA showed *KRAS* mutation rate slightly higher among African American compared to Caucasians (47% versus 43%) [43]. Studies performed in Latin America report *KRAS* mutation prevalence ranging from 13% (Colombia and Venezuela) to 40% (Chile) [44]. The European data showed mutation rate in Greece (29%), Netherland (37%), Germany (39%) and Slovenia (46%), Spain (48%) [43, 45, 46]. In Turkey, the mutation frequency for the *KRAS* mutation gene has been reported to be in the range of 11% to 49.1% [47–51]. Turkey is known from the geographical location between Europe, the Middle East and the Caucasus region. Thus, Turkey is comprised of many ethnic groups with European, Middle Eastern, Caucasian or Asian origins. This difference can be primarily attributed to ethnicity [51]. The *KRAS* mutation prevalence may also vary in the same population. Studies from Japan with homogeneous ethnic groups found a wide range of *KRAS* mutation frequencies (9–71%) [52, 53]. Studies with heterogeneous ethnic groups, such as studies from USA, also reported various frequencies of *KRAS* mutation (14–83%) [54, 55]. The finding of novel *KRAS* and *BRAF* gene mutations in cancerous tissue obtained from Saudi CRC patients were typical of those observed elsewhere, and may be attributed to environmental factors and/or racial/ethnic variations due to genetic differences in Saudi Arabia [56, 57].

In agreement with COSMIC, G12D and G12V mutation frequencies were the most frequent mutations among all CRC patients with *KRAS* mutation in North Africa. However, there are important differences among North Africa populations. In Morocco and Tunisia there is a higher prevalence of G12D mutation (≈50%) [22, 27]. G12D mutation accounts for about 41% of all the G12 mutations [58, 59]. Generally, *KRAS* mutations are predominantly found in codon G12 and mutations in codon 12 diminish both inherent and GAP-mediated hydrolysis without affecting the rate of nucleotide exchange, except for G12C, which exhibits GTPase activity similar to that of wild type [60]. G12C has become a promising target for novel strategies to treat *KRAS*-mutant CRC [61], adding up a new role for routine *KRAS* testing. A series of novel inhibitors that act against G12C in its GDP-bound state have been developed (such as Sotorasib and Adagrasib) and provides new therapeutic strategies to improve patient outcomes.

The aspects that seem to play an important role in the incidence of *KRAS* gene mutations in CRC in North Africa populations are the change in dietary patterns and nutrient intakes. In Morocco, The major pattern of nutritional change includes a large increase in the consumption of high calorie diets and fatty foods. Consumption of high amounts of these foods was associated with increased body weight, BMI, and risk of overweight and obesity. Between 2001 and 2014, the consumption of high-fat diets increased following the intake of oils which increased on average by 5.4 L (22.4 L against 17.02 L per person per year) [62]. Significant associations were found between the highest intakes of red meats, cold meats, sausages and the risk of CRC in a case–control study on dietary risk factors for CRC in Morocco [63]. High levels of animal protein, acrylamide foods, and low levels of vitamin A consumption have been shown to be associated with increased risk of CRC tumors with *KRAS* mutations [64]. Polyunsaturated fatty acids (PUFA) may be positively associated with CRC risk by potentially generating G > A transitions in the *KRAS* oncogene in Moroccan population. El Asri et al., showed that an increase in the consumption of PUFA above 16.9 g/day was associated with an increase in the presence of *KRAS* mutations (Odds Ratio OR = 2.48, 95% Confidence Interval CI = 1.22–4.96) as compared to the reference group whose consumption was less than 16.9 g [65]. A high intake of PUFA, in particular linoleic acid, may be an important dietary risk factor for *KRAS* mutated colon tumors, possibly by generating G > A transitions or G > T or G > C transversions in the *KRAS* oncogene [66]. S Deoula et *al*., showed that consumption of red meat was positively associated with colon cancer (OR = 1.23, 95% CI = 1.05–1.44) and CRC risk in Moroccan patients (OR = 1.14, 95% CI = 1.02–1.27) [67]. In Tunisia, the CRC incidence has increased markedly from 1994 to 2009, and it is suggested that if no interventions are implemented it is going to double by 2024. It is relevant to highlight that an upward trend in obesity largely explains the increasing incidence of CRC in Tunisian population. The trend of the incidence of obesity had increased from 10.9% in 1998 to 26.9% in 2016 [68] and 80% of Tunisian aged over 15 years old did not consume enough fruits and vegetables per day [69]. A high total day meat consumption (> 100 g) was significantly associated with a high risk of CRC compared to low consumption (< 50 g) in Tunisian population [70]. The growth of meat consumption is likely to increase in Morocco and Tunisia. Between 1971 and 2020, total production of meat of Morocco grew substantially from 205,387 to 1.45 million tonnes rising at an increasing annual rate that reached a maximum of 23.12% in 1987 and then decreased to 2.70% in 2020. Production of meat of Tunisia increased from 19,400 thousand tonnes in 1971 to 41,600 thousand tonnes in 2020 growing at an average annual rate of 2.19%. In Algeria, occupational exposures showed a significant link with an increased risk of CRC, as did obesity, alcohol consumption, and passive smoking. Yogurt, cereals, sugar, butter, and margarine consumption were significant protective factors, while cheese, dried fruits, red meat, juice, and fizzy drink consumption was associated with increased CRC risk [71]. The World Health Organization (WHO) and International Agency for Research on Cancer (IARC) declared that over-intake of red meat and/or processed meat increases CRC risk [72]. A recent Global Health Data Exchange review indicated that red and processed meat intake accounts for 1.77% and 1.18%, respectively, of worldwide CRC mortality, respectively [73]. The World Cancer Research Fund International Continuous Update Project showed that red meat or processed meats and alcohol consumption increase CRC risk and that food containing dietary fibre and dairy products decrease the CRC risk [74].

Red meat consumption may increase colon cancer risk by inducing the endogenous production of N-nitroso compounds and their precursors, which may induce *KRAS* mutations [70, 71]. Analysis of 900 CRCs with whole exome sequencing and epidemiologic annotations revealed an alkylating mutational signature that was associated with red meat consumption and distal tumor location, as well as predicted to target *KRAS* G12D/G13D [72]. Heterocyclic amines (HCAs) and polycyclic aromatic hydrocarbons (PAHs) formed during high-temperature meat cooking are also an important pathway for environmental colon cancer [73, 74]. HCAs and PAHs have been found to be mutagenic after they are metabolized by specific enzymes in the body. Previous studies have identified that the activity of these enzymes may be relevant to the cancer risks associated with exposure to these compounds [75, 76].

Interestingly, the use of antibiotics such as Fluoroquinolone and the third-generation cephalosporins was increasing in North Africa [77]. An increasing of antibiotic consumption was associated with an increasing CRC risk, particularly when used commonly [78] and colon cancer pathogenesis across all age-groups in a large population-based case–control study of patients with early onset-CRC [79]. Antibiotics are known to influence the gut microbiome [80, 81], which has been implicated in CRC or tumor progression, possibly through bacterial involvement in nutrient metabolism and direct interaction with gut mucosa [82]. In the context of CRC, bacteria have been shown to play a role in cell signaling [83–85]. In a *KRAS*-specific context, the role of *KRAS* signaling on the tumor microbiome is still to be determined, even though, it is accepted that the exposure to bacterial can shape the development of CRC of which *KRAS* can be one major player [86, 87]. Nevertheless, in *KRAS*-driven CRCs, it has been shown that genotoxic stress and some other factors, including metabolites produced by the microbiota, can facilitate genetic and epigenetic changes leading to carcinogenesis [88]. A priori, computational modeling is a promising approach for studying genotype-to-metabolic-phenotype relations or microbe–microbe and host–microbe metabolic interactions in CRC [89]. Microbiome modulation is one of the most prospective new strategies in medicine to improve the gut health in individuals and is likely to play a key role in the outcome of colorectal surgery in North Africa.

Modifiable dietary and lifestyle risk factors could prevent most cases of CRC and. Up to 47% of CRC cases could be prevented by staying physically active, maintaining a healthy body weight and eating a healthy diet [90]. In North Africa, dietary and lifestyle factors are one of the most promising approaches to reduce the occurrence and progression of CRC. Dietary and lifestyle factors may not only play a role in causing mutations and epigenetic modifications, but also in enhancing tumor growth in tissues that have already acquired specific epigenetic aberrations. There may be direct causal associations between diet and lifestyle factors and molecular changes in CRC, and establishing this is important for prevention strategies, and increasing the ability to better predict disease progression and prognosis [91].

Beside established anti-CRC treatment, better understanding of the causality of CRC can be established by combining epidemiology and genetic/epigenetic on CRC etiology. This approach may be able to significantly reduce the burden of disease in North Africa population. Furthermore, the government should develop policy on CRC prevention and public health programs which may serve as a feasible setting to increase public awareness on lifestyle risk factors.

## Data Availability

The datasets generated and analyzed during the current study are available in the [Prevalence and Patterns of Mutations in RASRAFMEKERKMAPK Signaling in North Africa Tables] repository.Pub med (Open access): https://pubmed.ncbi.nlm.nih.gov/. Science Direct (Open access): https://www.sciencedirect.com/. Google Scholar (Open access): https://scholar.google.com/.

## Abbreviations

ASCO: American Society of Clinical Oncology
ASR: Age-Standardized Incidence Rates
BMI: Body Mass Index
CI: Confidence Interval
COSMIC: Catalogue Of Somatic Mutations In Cancer
CRC: Colorectal Cancer
EGFR: Epidermal Growth Factor Receptor
ERK: Extracellular-signal-Regulated Kinase
GDP: Guanosine Bi-Phosphate
GLOBOCAN: Global Cancer Observatory: CANCER TODAY
GTP: Guanosine Tri-Phosphate
HCAs: Heterocyclic Amines
HDI: High Human Development Index
HRAS: Harvey Rat Sarcoma
IARC: International Agency for Research on Cancer
KRAS: Kirsten Rat Sarcoma
MAPK: Mitogen-Activated Protein kinase
MEK: Mitogen-Activated Protein kinase kinase
NRAS: Neuroblastoma Rat Sarcoma
OR: Odds Ratio
PAHs: Polycyclic Aromatic Hydrocarbons
PUFA: Polyunsaturated fatty acids
RAF: Rapidly Accelerated Fibrosarcoma
RAS: Rat Sarcoma
SSA: Sub-Saharan Africa
UAE: United Arab Emirates
USA: United States of America
WHO: World Health Organization
